# Development and validation of rapid environmental DNA (eDNA) detection methods for bog turtle (*Glyptemys muhlenbergii*)

**DOI:** 10.1371/journal.pone.0222883

**Published:** 2019-11-14

**Authors:** Anish A. Kirtane, Maxwell L. Wilder, Hyatt C. Green

**Affiliations:** SUNY-ESF, Department of Environmental and Forest Biology, Syracuse, NY, United States of America; University of Hyogo, JAPAN

## Abstract

Bog turtles (*Glyptemys muhlenbergii*) are listed as Species of Greatest Conservation Need (SGCN) for wildlife action plans in every state it occurs and multi-state efforts are underway to better characterize extant populations and prioritize restoration efforts. However, traditional sampling methods can be ineffective due to the turtle’s wetland habitat, small size, and burrowing nature. Molecular methods, such as qPCR, provide the ability to overcome this challenge by effectively quantifying minute amounts of turtle DNA left behind in its environment (eDNA). Developing such methods for bog turtles has proved difficult partly because of the high sequence similarity between bog turtles and closely-related, cohabitating species, most often wood turtles (*Glyptemys insculpta*). Additionally, substrates containing bog turtle eDNA are often rich in organics or other substances that frequently inhibit both DNA extraction and qPCR amplification. Here, we describe the development and validation of a qPCR assay, BT3, targeting the mitochondrial cytochrome oxidase I gene that correctly identifies bog turtles with 100% specificity and sensitivity when tested on 201 blood samples collected from six species over a wide geographic range. We also developed a full-process internal control employing a genetically modified strain of *Caenorhabditis elegans* to improve DNA extraction methods, limit false negative results due to qPCR inhibition, and measure total DNA recovery from each sample. Using the internal control, we found that DNA recovery varied by over an order of magnitude between samples and likely explains the lack of bog turtle detection in some cases. Methods presented herein are highly-specific and may offer a more cost effective, non-invasive tool to supplement bog turtle population assessments in the Eastern United States. Poor or differential DNA recovery, which remains unmeasured in the vast majority of eDNA studies, significantly reduced the ability to detect bog turtle in their natural environment.

## Introduction

The bog turtle (*Glyptemys muhlenbergii*) is a federally-threatened species listed as a Species of Greatest Conservation Need (SGCN) in every state it occurs and is listed as critically endangered by IUCN[1–3]. It is distributed in small populations along the Eastern US ranging from Northern Georgia to New York [1,2,4,5]. Due to significant urban growth and new construction of energy and transportation infrastructure encroaching on their habitat, there is an urgent need to survey and prioritize specific populations for protection and management [6,7]. However, due to their small size and burrowing nature [5,8], currently available methods to detect and enumerate bog turtles through trapping and probing are highly resource intensive and, in some cases, ineffective[7]. Alternative methods that reliably detect bog turtles without requiring a tremendous amount of resources are needed to properly assess the status of this endangered species.

Like all animals, turtles shed small amounts of DNA into the environment (eDNA) through feces, skin cells, and other secretions[9], the detection or quantification of which can be used as a proxy for the presence of cryptic species that are difficult and resource intensive to detect otherwise[10–13]. eDNA methods have been used previously to determine the presence of a wide range of aquatic species[10,11] and can be 2–10 times more cost-effective than traditional surveying methods for freshwater turtles[14]. While sequencing-based metagenomics (i.e., metabarcoding) studies are useful for estimating the relative abundance of a wide taxonomic range of species[12,15,16], quantitative PCR (qPCR) detection methods usually target a single species and offer unmatched sensitivity—fitting for studies where intact target eDNA fragments are thought to be rare[17–20].

The prospect of bog turtle detection with eDNA poses a unique combination of challenges: 1) individuals are small and are often found in small, sparse populations reducing the amounts of eDNA expected to be released into the surrounding environment even when abundant[4], 2) the bog habitat is rich in sediments and organics, such as humic acids, that can interfere with DNA extraction and qPCR amplification [21–23], 3) much of the shed DNA is hypothesized to be both diluted and exported off-site via small streams and rivulets, and 4) the wood turtle (*Glyptemys insculpta*), a genetically-related species [24], often cohabitates with bog turtles introducing the possibility for cross-reaction via non-specific PCR amplification.

In this study, our goal was to develop a method that could overcome these challenges to reliably detect the presence of bog turtles in their native environment. First, we amassed a blood and tissue reference DNA collection composed of 201 samples collected from six species across 10 states representing the extant geographic range of bog turtle. Species-specific qPCR oligonucleotides targeting mitochondrial cytochrome oxidase 1 gene (COI) were designed *in silico* and tested for sensitivity and specificity using the reference DNA collection. Then, we developed a full-process internal control and a qPCR assay targeting an artificial green fluorescent protein (*gfp*) gene in genetically modified *Caenorhabditis elegans* to improve DNA recovery methods and to indicate particularly difficult-to-extract bog samples. The qPCR assays were applied to both contrived samples (15cm from a bog turtle), providing opportunities for methodological optimization in an ideal scenario, and non-contrived samples as a proof-of-principle. While we were able to use the BT3 assay to detect bog turtles in their natural environment somewhat successfully, significant sample-to-sample variability in DNA recovery was observed and may underscore the need to integrate measurements of DNA recovery into studies that rely on quantifying or detecting eDNA in complex environmental samples.

## Materials and methods

### Reference sequences and primer design

Species-specific primers and probes were designed to target the cytochrome oxidase subunit 1 (COI) gene sequence based on recommendation of previous studies[9]. Bog turtle sequences were aligned with closely-related and co-occurring species with MAFFT [25] using sequences obtained from GenBank (S1 Table). Oligonucleotides BT3F, BT3R, and BT3P targeting bog turtle (Table 1) were designed manually to target regions with high sequence similarity among bog turtle sequences and high mismatch with non-target species sequences. *C*. *elegans* primers (CG4F, CG4R) and probe (CG4P) were designed to target the artificial *gfp* gene in the SH52 strain (S1 Text) used as an internal control. Blastn[26] analysis of each oligonucleotide confirmed that no sequences in the Genbank nr database (version 233.0, released August 15, 2019), aside from their intended targets, matched all three oligonucleotides.

**Table 1:** Oligonucleotide sequences designed in this study.

Oligonucleotide name	Sequence (5’-3’)	Final reaction concentration (nM)	Amplicon size (bp)
BT3F	GGAGTCGAAGCAGGAGCG	1400	71
BT3R	CCGGCGTGGGCCAG	1400	
BT3P*	[FAM]ACA GGC TGA[ZEN]ACT GTA TAC CCT CCA CTA GCC G[IBFQ]	100	
CG4F	CGA AAG ATC CCA ACG AAA AGA GAG	1400	73
CG4R	CCA TGT GTA ATC CCA GCA GCT	1400	
CG4P**	[VIC]ACA TGG TCC TTC TTG AGT TTG TAA C[MGB]	100	

*internal ZEN^™^ quencher probe obtained from IDT (Coralville, IA)

**minor-groove binder probe obtained from ABI (Waltham, MA)

### Reference DNA collection

Reference DNA was composed of DNA extracted from blood and tissue samples collected throughout the known bog turtle range[27] under the purview of the respective state agencies. The reference library was analyzed for candidate genetic markers and allowed estimations of assay sensitivity (true positives/# of bog turtle samples tested X 100) and specificity (true negatives/# of non-bog turtle samples tested X 100) across the broader population (Table 2).

**Table 2:** Taxonomic and regional distribution of blood and tissue reference samples.

	*Clemmys guttata*	*Emydoidea blandingii*	*Glyptemys insculpta*	*Glyptemys muhlenbergii*	*Terrapene carolina*	*Chelydra serpentina*	Total
Connecticut	1	-	1	4	2	-	8
Georgia	-	-	-	10*	-	-	10
Massachusetts	-	10	7	-	-	-	17
Maine	-	-	6	-	-	-	6
New Hampshire	-	-	5	-	-	-	5
New Jersey	-	-	-	43	-	-	43
New York	3	-	2	29	-	-	34
Pennsylvania	3	-	5	49	1	1	59
Tennessee	-	-	-	9*	-	-	9
Virginia	-	-	-	10*	-	-	10
**Total**	**7**	**10**	**26**	**154**	**3**	**1**	**201**

*tail clipping tissue samples. All others are blood samples.

DNA was extracted from 20 μl of buffered blood using the Qiagen Blood & Tissue DNA Extraction Kit (Valencia, CA). Blood from only one species was extracted on any single day to limit the possibility of cross-contamination. Tail clipping DNA extracts from GA, TN, and VA were extracted during a previous project using the same extraction method.

### qPCR conditions

TaqMan^®^ qPCR reactions consisted of 12.5 μl of 2X TaqMan Environmental Master Mix (version 2.0), oligonucleotides at the appropriate concentrations (Table 1), 2 μl template DNA, and molecular grade water (Invitrogen, #10977–015). SYBR Green qPCR was used for early assay optimization and was prepared in the same manner as probe-based qPCR reactions except the probe was excluded and 0.25 μl of 10X SYBR Green (diluted from 10,000X in TE buffer) was added for a final concentration of 0.1X SYBR Green as done previously[28]. All further mentions of qPCR assays refer to their probe-based versions. Serial dilutions of both synthetic dsDNA gBlocks^®^ and tissue DNA extracts were used to assess assay kinetics while standard curves generated using the gBlock were used to calculate copy number. The gBlock contained sequences for both amplicons, BT3 and CG4 (297bp, S1 Fig). All oligonucleotides were obtained from IDT (Coralville, IA) except for the minor groove binder probe, CG4P, which was obtained from ABI (Waltham, MA). All qPCR reactions were run in triplicate in 25 μl volumes on a QuantStudio3 (ABI) under default thermocycling conditions: 50°C for 2 minutes, 95°C for 10 minutes, and 40 cycles of 95°C for 15 seconds and 60°C for 1 minute. Prior to export and data analysis, the ‘automatic’ setting was used to define baseline fluorescence of each reaction and the fluorescence threshold was manually set to 0.03 for all reactions.

### Full-process internal control

A genetically modified strain of *C*. *elegans* (SH52) [29] whose genome contains a high copy number of a genetically modified *gfp* gene was used to estimate both DNA extraction recovery and qPCR inhibition simultaneously similar to methods described previously [22]. While other organisms have been used to control for DNA loss and PCR inhibition[30,31], we chose *C*. *elegans* to serve as an internal control because 1) it is a model organism that can be grown, stored, observed, and genetically manipulated easily using standard protocols, 2) its eukaryotic membrane may mimic DNA extraction from shed bog turtle cells more closely than bacterial[22] or viral[31] internal controls used previously, and 3) it is eutelic which facilitates the collection and processing of a known number of cells. *C*. *elegans* was grown on an *E*. *coli* strain OP50 lawn on NGM (Nematode Growth Media) at 25°C for at least 7 days before use[32]. Briefly, ~50–70 adult hermaphrodite worms were added to a 2 ml tube with 1.4 mm ceramic beads (VWR #10158–610, Radnor, PA) and 1 ml of elution buffer (GeneRite, North Brunswick, NJ). Worms were homogenized for 40 sec at 6 m s^-1^ on a FastPrep-24 5G (MPBio, Santa Ana, CA) to break apart worms to provide a lysate consisting of a mixture of free DNA and cell matter and dispensed into 50 μl aliquots. Lack of complete lysis was confirmed by the presence of some remaining tissue particles using DAPI staining and fluorescent microscopy (S2 Fig). Aliquots of *C*. *elegans* lysate were stored at -20°C before spiking samples. Two microliters of lysate were also used directly as template in CG4 qPCR to quantify the number of *gfp* gene copies spiked and the number of gene copies that correspond to a theoretical 100% recovery accounting for the fact that only two of 100 ul of eluted DNA was used in each qPCR reaction. The amount of *C*. *elegans* lysate spiked was such that approximately a 1% DNA recovery would still result in quantifiable level of spike and the generation of an amplification curve that could be used for detection of qPCR inhibition (see “qPCR Quality Control and Assurance” below). Percent recovery of the spike was then calculated which was used as a correction factor for BT3 marker abundance (Equations A, B and C in S1 Text). Thus, estimation of DNA recovery permitted not only the correction for BT3 marker loss during DNA extraction, but also various optimization experiments such as those used to determine optimal lysing matrix (S3 Fig) and sample collection/processing methods (S4 Fig).

### Environmental sample collection and processing

Environmental samples were collected when bog turtle activity is thought to be highest between mid-May and early-June in both 2017 and 2018. In 2017, bog samples were collected from two sites in New York, US (NY-14-34A and NY-38-03) and one in Pennsylvania, US (DE-PA-11A) with known bog turtle populations (Table 3). Samples were also collected from a site in Southern New York (NY-14-14) where bog turtles have not been observed despite multiple attempts over the last 20 years. For optimization purposes, 24 contrived samples were collected in the vicinity (within 15 cm) of a visible bog turtle. These contrived samples also provided ideal scenarios for methodological optimization. For 2017 samples, water and suspended sediment was collected in sterile one-liter bottles, stored on ice, and processed within eight hours of sampling. To determine whether eDNA was more concentrated in solution or bound to sediment, 500 ml of each sample was distributed between 10 50 ml screw-cap tubes while being stirred continuously using magnetic stir bars to keep the sediment suspended (S5 Fig). Freshly prepared *C*. *elegans* lysate corresponding to approximately 1.5x10^5^-2.5x10^5^ *gfp* gene copies was added to each of the 10 falcon tubes. The 50 ml tubes were shaken by hand for 10 s to distribute spike and centrifuged at 2,000 g for 10 min. Without disturbing the sediment pellet, the supernatant from all 10 tubes was filtered through 0.4 μm pore-size 47mm diameter polycarbonate filters until water from all 10 tubes were filtered or until three filters (per sample) were clogged. In the latter case, the filtered volume was noted. Filters were placed directly in bead tubes and stored at -80°C until extraction. Sediment pellets ranging from 0.9 to 7.4 g per falcon tube were stored at -80°C until DNA extraction.

**Table 3:** Sample distribution in 2017 and 2018.

Site code*	Recent BT presence?	n samples (2017)**		n samples (2018)***	
		Contrived	Unknown	Contrived	Unknown
NY-14-01	Yes	0	0	2	5
NY-40-01	Yes	0	0	2	5
NY-14-34A	Yes	5	0	2	5
NY-14-14	No	0	4	0	0
NY-14-16B	No	0	0	0	5
DE-PA-11A	Yes	1	3	3	5
DE-PA-38	Yes	0	0	3	4
DE-PA-28	Yes	0	0	3	4
DE-PA-17	No	0	0	0	3
LP-NY-38-03 (Oswego)	Yes	3	0	0	0
Total		9	7	15	36

*Precise site locations are masked to maintain confidentiality.

**In 2017, water and suspended sediment were collected.

***In 2018, non-suspended sediment, sometimes containing vegetation were collected.

In 2018, six new sites were sampled in addition to revisiting two of four sites from 2017 sampling. Also in 2018, water was avoided and non-suspended sediments and sometimes vegetation were collected. Ten to twenty five grams of non-suspended sediment was collected from areas in or around known or suspected hibernacula. Sediment was collected in a 50 ml screw-cap tube, sealed in a WhirlPak^®^ bags (Nasco, Fort Atkinson, WI), stored at -4°C, then shipped to Syracuse, NY for DNA extraction and long-term storage at -80°C. For 2018 samples only, *C*. *elegans* lysate corresponding to 9.9x10^3^-2.7x10^4^ *gfp* gene copies was added to approximately 500 mg of sediment in lysing matrix C tubes (MPBio, Santa Ana, CA) immediately before extraction.

### DNA extraction from environmental samples

DNA from filters was extracted using DNA-EZ kit (GeneRite, North Brunswick, NJ) following manufacturer’s protocol. Sediment was extracted using FastDNA^TM^ SPIN Kit for Soil (MPBio, Santa Ana, CA) homogenizing for 20 sec at 6 m s^-1^ using lysing matrix C based on consistent performance in extraction trials under these conditions (S3 Fig). For each sediment sample, we combined 25 μl eluate from four replicate extractions to yield 100 μl total elution volume per sediment sample. Extraction blanks were added to each batch of extractions and were performed in the same manner as environmental samples but without adding sediment or filters. Concentration of eluted DNA was assessed on a Qubit 3.0 fluorometer (ThermoFisher, Wilmington, DE) following the manufacturer’s protocol.

### Data interpretation at low template concentrations

Samples in which all three replicate BT3 Ct values were less than the Ct value of the average gBlock limit of quantification (LOQ) (31.604) were considered within the range of quantification (‘quantifiable’) and converted to copy number using the assay-specific gBlock standard curve equation. Samples in which any triplicate wells did not amplify sufficiently to cross the threshold before 40 cycles were considered below the LOD. In a liberal interpretation of the data, samples were considered within the limit of detection (LOD), but not within the LOQ if any of the three replicate Ct values were between 31.604 and 40 (‘detectable, but non-quantifiable’). The absence of amplification from all NTCs and sample processing blanks permitted such an interpretation of high Ct values. Samples within the LOD are herein referred to as ‘detectable’.

### qPCR quality control and assurance

All qPCR reactions were performed with a unidirectional workflow and separation of workspace under dedicated PCR or dead-air hoods. That is, reaction building, consisting of oligonucleotide and enzyme mixing and distribution across wells, occurred separate from template addition and amplicon production. The absence of qPCR inhibition in BT3 and CG4 amplification profiles was confirmed by applying kinetic outlier detection (KOD) methods described previously[22]. Briefly, all amplification profiles were fit with a four parameter model after log-transformation and first (*t*_*1*_) and second derivative maxima (*t*_*2*_) were calculated from the fitted curve. Then, using standard reactions as a reference, the linear relationship between *t*_*1*_ and *t*_*2*_ was determined. Finally, all environmental sample amplification profiles were searched for kinetic outlier reactions where the relationship between *t*_*1*_ and *t*_*2*_ differed significantly from that of the reference (un-inhibited) reactions (*τ_norm_* < -3.84 [95% chi-squared probability with one degree of freedom]). No amplification was observed in sample processing blanks (n = 32) or no-template qPCR controls (NTCs, n = 75) throughout the study.

## Results

### Assay amplification kinetics

BT3 and CG4 assays, targeting bog turtle and *C*. *elegans* respectively, showed a consistent amplification efficiency of >98%, R^2^ of ≥0.995±0.005, and limits of detection at ≤10 copies (Table 4) on synthetic template standard curves. Comparable performance characteristics on tissue DNA extracts suggest that the assays operate similarly within their native genomic context. In some cases, gel electrophoresis of amplicons showed the presence of some non-specific amplification on bog turtle blood DNA (S6 Fig). Multiple unsuccessful attempts were made to eliminate this non-target amplification despite the fact it did not seem to affect qPCR amplification kinetics or assay sensitivity/specificity and was observed only in bog turtle blood DNA and not in other species tested.

**Table 4:** BT3 and CG4 assay performance characteristics on synthetic template (gBlock) and tissue DNA extract dilutions. Blood and whole worms were used as starting material for tissue DNA extracts for the BT3 and CG4 assays, respectively.

	gBlock			Tissue DNA extracts		
Assay	Cal. Eq.	Eff.	LOQ (copies)	Cal. Eq.	Eff.	LOQ (fg DNA)
BT3	y = -3.327x+34.931	0.997	10	Y = -3.326x+39.002	0.998	95
CG4	y = -3.374x+39.439	0.998	10	Y = -3.370x+41.670	0.9803	9.4

### Assay specificity and sensitivity

Blood samples yielded on average 2.8±2.8 and 4.8±2.8 ng μl^-1^ DNA for bog turtle and non-bog turtle samples, respectively. Tail-clipping DNA extracts yielded on average 23.0±27.4 ng μl^-1^. After screening all 201 blood and tissue extracts with the BT3 assay, all 154 bog turtle samples exhibited strong amplification whereas amplification did not occur with any of the 47 non-bog turtle samples. Bog turtle blood DNA and tail clipping DNA contained an average of 1.3x10^6^ ±6.6x10^6^ and 2.9x10^2^ ±5.4x10^2^ BT3 copies/ng DNA, respectively. The high variability of BT3 copies per ng DNA may reflect some variability in the number of mitochondria per cell or the age of the sample.

### Results of sample processing optimization trials

Sample processing optimization trials performed using a single sample from site DE-PA-11a suggested that higher quantities of eDNA were recoverable from sediment compared to water (S4 Fig) and that homogenization for 20 seconds using lysing matrix C provided consistently high DNA recovery (S3 Fig).

### Detection of bog turtle eDNA from contrived samples

Lower than expected detection rates in contrived samples (54%) collected over the entire study period point to the paucity of detectable bog turtle eDNA in their environment (Table 5). However, significant differences in detection and quantification rates in contrived samples between years and their respective sampling approaches provided some indication of how eDNA was distributed throughout the system. In 2017, zero contrived water samples had quantifiable eDNA, while 66% had detectable, non-quantifiable levels reflecting dilution and diffusion throughout the immediate sampling area. In contrast, 40% of 2018 contrived non-suspended sediment samples contained quantifiable eDNA, while only one sample (6%) had detectable, non-quantifiable eDNA suggesting that eDNA remained at high concentrations but only near the initial points of contact with sediment.

**Table 5:** Detection of bog turtle eDNA in contrived 2017 and 2018 samples. Contrived samples were collected within about 15 cm of a bog turtle. Percent recovery indicates the recovery of DNA from each sample measured using the internal control.

Site	# Marked/ha *	Matrix**	Num. Samples	Mean % Recovery of *C*. *elegans* spike (Std. Dev.)	Quantification Rate (Within LOQ Only)	Detection Rate (Within LOD)***
DE-PA-11A	127.7	WS	1	11.3 (11.3)	0	1
DE-PA-11A	127.7	NSS	3	54 (12.2)	0.67	0.67
DE-PA-28	53.3	NSS	3	52.4 (14.1)	0.33	0.67
DE-PA-38	4.7	NSS	3	39.9 (12.1)	0.33	0.33
LP-NY-38-03	9.86	WS	3	10.43(1.8)	0	0.67
NY-14-14	2.2	WS	5	11.6 (9.5)	0	0.6
NY-14-34A	2.2	NSS	2	23.4 (8.2)	0	0
NY-14-01	30	NSS	2	22.4 (12.9)	1	1
NY-40-01	5	NSS	2	33.4 (10.2)	0	0

* # Marked/ha refers to the number of bog turtles found in each of the field sites during the an ongoing monitoring program (based on personal communication with Lori Erb, MACHAC).

**NSS = Non-suspended sediment; WS = water with suspended sediment

***Includes samples within LOQ

When in the quantifiable range, BT3 marker copies in contrived samples ranged from 1.6x10^3^ to 1.3x10^5^ per gram after accounting for sample-specific recoveries. Sample- specific recoveries were used to account for eDNA loss during extraction using recoveries of the *C*. *elegans* spike. Average DNA recovery was also higher in 2018 contrived samples when sediment was directly sampled (37.4±17.3) compared to 2017 when sediment in the water samples was pelleted (12.1±7.4; one-tailed t-test, *P* <0.001), which may have aided detection/quantification. Importantly, not accounting for DNA recovery would have provided underestimates of marker concentration ranging from 0.09 to 0.54 times their actual values.

### Detection of bog turtle eDNA from non-contrived samples

eDNA detection rates fell significantly when turtle presence in the immediate sampling area was unknown, presumably due to the absence of turtles from specific locations or the decay of eDNA over time. Eighty six percent of samples were below the LOD for BT3 markers. eDNA was detected in only two of the six sites known to be inhabited and, in those sites, detection rates ranged from 20–75%. Only one sample from each year had quantifiable eDNA (both from the densely populated site DE-PA-11a). In resampled sites, NY-14-16B and DE-PA-11a, detection rates fell slightly in 2018 despite an increased sampling effort and higher DNA recoveries (Tables 5 & 6). Surprisingly, bog turtle eDNA was also detected at the 2017 extirpated site, NY-14-16B, which suggests the eDNA method may be useful in identifying previously unidentified bog turtle populations despite overall low detection rates. Re-extraction and re-analysis of this sample confirmed the previous result.

**Table 6:** Detection of bog turtle eDNA in non-contrived 2017 and 2018 samples.

Site	#Marked/ha*	Matrix**	Num. Samples	Mean % Recovery of *C*. *elegans* spike (Std. Dev.)	Quantification Rate (Within LOQ only)	Detection Rate (Within LOD)***
DE-PA-11A	127.7	WS	3	11.3 (11.3)	0.33^	0.33
DE-PA-11A	127.7	NSS	5	54 (12.2)	0.2	0.2
DE-PA-17	0	NSS	3	26.7 (4.4)	0	0
DE-PA-28	53.3	NSS	4	52.4 (14.1)	0	0
DE-PA-38	4.7	NSS	4	39.9 (12.1)	0	0.75
NY-14-34A	2.2	NSS	5	23.4 (8.23)	0	0
NY-14-01	30	NSS	5	22.4 (12.9)	0	0
NY-14-16B	0	WS	4	13.6 (6.8)	0	0.25
NY-14-16B	0	NSS	5	61.3 (13.7)	0	0
NY-40-01	5	NSS	5	33.4 (10.2)	0	0

* # Marked/ha refers to the number of bog turtles found in each of the field sites during the an ongoing monitoring program (based on personal communication with Lori Erb, MACHAC)

**NSS = Non-suspended sediment; WS = water with suspended sediment

***Includes samples within LOQ

^The sample with quantifiable marker concentrations was the only non-suspended sediment sample from this site in 2017. The other two samples collected from this site in 2017 were water with suspended sediment.

DNA recoveries among all non-contrived samples ranged from 1.3 to 74.3% and showed no clear pattern in relation to sampling site or sample appearance. Recoveries from DE-PA-28 (54.1%) were significantly higher than NY-14-01 (13.6%; Tukey HSD, *P* < 0.05). All other pairwise site recoveries were statistically insignificant. Mean DNA recovery was 29.2% lower in 2017 non-contrived samples compared to 2018 (one-tailed t-test, *P* < 0.001). Quantifiable marker concentrations were found only in non-contrived samples with DNA recoveries ≥22%; however, eDNA was found in samples with low recovery (as low as 7.0%) and not found in samples with high recovery (as high as 74.3%) suggesting other factors also determine detection rates.

## Discussion

The newly-developed bog turtle eDNA methods permitted an average of 15.3% detection rate average across sites and a range of 0–75% site detection rate indicating that under the right conditions these newly developed methods may supplement or improve the effectiveness of existing techniques. Despite high detection rates in some sites, a significant limitation to the developed methods is the high rate of false negative results (50% when calculated at the site level). A number of factors could have contributed to our relative inability to detect BT3 markers when bog turtles were present on site. Based on the marker’s prevalence in all bog turtles tested and on the moderate DNA recoveries (most >20%) from the majority of non-contrived samples, it’s unlikely that primer-template mismatches or poor DNA recovery are solely responsible for high false negative rates.

Suspended cells or other eDNA-containing particles are often concentrated on polycarbonate or glass fiber filters[14,33,34], which offer practical advantages over eDNA extraction from sediment. However, direct filtration at all sites was unfeasible in this study due to surface waters either being non-existent or extremely rich in organics which clogged filters almost immediately. Furthermore, when filtration was possible, it captured undetectable amounts of eDNA relative to that obtained from sediment for both bog turtle contrived samples and *C*. *elegans* internal control spike. Other studies also demonstrated that eDNA is more concentrated and persistent in sediment than in surface water[35] and proposed that DNA in the adsorbed phase might remain protected from microbial and enzymatic degradation[36,37]. This further highlights the potential for sediments to store eDNA at relatively high concentrations and possibly serve as a more useful sampling matrix for eDNA studies where filterable water may not be available[35].

Using a novel internal control we found that the recovery of total DNA from sediment samples varied by over an order of magnitude. In some cases, DNA recovery differed greatly between samples of indistinguishable composition taken within the same site highlighting the unpredictability of difficult-to-extract samples in these types of environments as well as the need for such controls to prevent incorrect interpretation of false negative samples. KOD analysis showed the absence of significant qPCR inhibition, thus attributing the variability in the recovery of the internal control spike to loss during sample process and DNA extraction. eDNA studies that seek to make quantitative inferences about the number of individuals[38,39]or species biomass[12,40] based on the quantity of eDNA markers should account for variable DNA recovery across samples.

DNA is known to rapidly diffuse from the source in aquatic systems and, where flux is relatively high in streams or rivulets, DNA is transported out of the system relatively quickly[34]. Furthermore, most eDNA-containing particles including skin cells settle out of slow-moving water into the sediment[33] where eDNA has been shown to persist longer than in surface water[35]. Our findings suggest that these dynamics may also be controlling the fate, transport, and storage of eDNA in bogs where eDNA is stored mostly in sediment in or near hibernacula and a targeted sampling approach incorporating prior knowledge of the site as well as animal behavior may lead to higher detection rates. Such dynamics would suggest eDNA suspended in water would be more indicative of recent bog turtle activity. It is impossible to tell with these methods what proportion of bog turtle eDNA was shed from live versus dead individuals and it is expected that carcasses store and shed DNA for long time periods[41]. Future studies aimed at quantifying decay rates of BT3 markers as a function of environmental factors (e.g., temperature, pH, redox conditions) are needed not only to develop more precise sampling strategies but also to enable quantitative population estimates of bog turtle using eDNA methods.

The development of eDNA methods for the detection of bog turtles from environmental samples may be a useful tool for managing the recovery of this species. Like eDNA studies of other rare aquatic species, such as the hula frog (*Latonia nigriventer*)[42], we found that eDNA methods provided some evidence of bog turtle presence at a site where traditional methods had been applied with little success. Incorporating eDNA analysis has been shown to improve traditional survey methods for many reptile and amphibian species including American bullfrog[20], eastern hellbender[10] and wood turtle[43] and eDNA methods are more easily scaled up facilitating cost-effective site screening[14,44] over a wide geographic range. While higher sample numbers per site and possibly larger sample sizes (e.g., > 2 g sediment) would be needed to overcome significant variability in detection of bog turtle, such an approach would enable prioritization of specific sites for follow-up assessment and potentially lead to the discovery of previously unknown sites.

## Supporting information

S1 Table Accession numbers of COI sequences used to build alignments and design oligonucleotides. (PDF)

S1 Text Supplemental methods and description of experimental trials. (PDF)

S1 Fig Oligonucleotide binding sites on gBlock used for standard curve generation. (PDF)

S2 Fig Micrograph of *C*. *elegans* lysate. Fluorescent micrograph of *C*. *elegans* lysate using DAPI stain (DNA-binding) showing intact cells and clumps of cells present in the lysate. Lysate used to spike in DNA extractions was filtered onto a 0.2 μm pore-size 25mm polycarbonate filter, stained, and viewed under a fluorescent microscope. The field of view is approximately 500 *μ*m wide. A partially intact *C*. *elegans* individual appears near the top of the image. Cellular debris of various sizes can also be seen in the image indicating the bead-beating procedure does not completely lyse all tissues.
(PDF)

S3 Fig Results from lysing matrix and bead beating time optimization trial. Lysing matrix C was selected for its consistently high recovery with 20 s bead-beating.
(PDF)

S4 Fig Results from sample matrix trial. Detectable DNA recovery from the sediment and supernatant water from field samples during extraction efficiency trials. “S1-S6” and “W1-W6” denote sediment and aqueous phases, respectively.
(PDF)

S5 Fig Illustration of eDNA and *C*. *elegans* internal control processing workflow for 2017 environmental samples. (PDF)

S6 Fig Gel image of non-specific amplification in BT3 assay. Gel electrophoresis of qPCR products from BT3 (A) and CG4 (B) on 2% agarose gel using 1X TBE buffer. **L.** 1kb Ladder; **Std.** gBlock standard; **Bt**. Bog turtle blood DNA; **11–1, OS-1, 34–4, 34–2.** Environmental sediment sample DNA with *C*. *elegans* spike; **Cg.** *C*. *elegans* tissue DNA; **UnS.** Sediment with no *C*. *elegans* spike.
(PDF)
