# Supplementary material for: Development and validation of rapid environmental DNA (eDNA) detection methods for bog turtle (Glyptemys muhlenbergii)
Source: PLoS One. 2019 Nov 14;14(11):e0222883. doi: 10.1371/journal.pone.0222883 (PMC6855662; doi:10.1371/journal.pone.0222883)
Supplement: S1 Table — (PDF) [file pone.0222883.s001.pdf]

S1 Table. Accession numbers of COI sequences used to build alignments and design oligonucleotides.

Species	Accession numbers
Glyptemys muhlenbergii	KC750824.1, KU985629.1, KX559036.1, HQ329645.1, KC181206.1
Glyptemys insculpta	KC750823.1 , KX559035.1 , KX559034.1 , HQ329644.1 , KC181207.1
Chrysemys picta	MH273650.1, MH273649.1, MH273648.1, MH273647.1, KC181209.1
Clemmys guttata	KU986128.1, KU985699.1, KU985630.1, KX559015.1, HQ329641.1
Emydoidea blandingii	KU985909.1, KU985853.1 , KU985825.1, KU985739.1
Actinemys marmorata	HQ329640.1
Deirochelys reticularia	KC181208.1
Emydoidea blandingii	HQ329642.1
Emys orbicularis	HQ329643.1
Malaclemys terrapin	HQ329654.1
Mauremys leprosa	AY337351.1
Terrapene carolina	HQ329658.1
Trachemys scripta	FR874840.1
