# Supplementary material for: Development and validation of rapid environmental DNA (eDNA) detection methods for bog turtle (Glyptemys muhlenbergii)
Source: PLoS One. 2019 Nov 14;14(11):e0222883. doi: 10.1371/journal.pone.0222883 (PMC6855662; doi:10.1371/journal.pone.0222883)
Supplement: S1 Text — (PDF) [file pone.0222883.s002.pdf]

S1 Text. Supplemental methods and description of experimental trials.

Calculating percent recovery using *C. elegans* full process internal control

The percent recovery for filters (R_f) and sediment (R_s) was calculated using the following equations where X_d is copies/μl *C. elegans* gfp gene copies detected in the environmental sample DNA extract, V_e is the total volume of environmental DNA eluate (μl), X_l is copies/μl of the *C. elegans* gfp gene copies spiked, V_s is the volume of *C. elegans* lysate spiked (μl) and M_i is the mass of sediment collected in the falcon tube and M_e is the mass of the sediment used in DNA extraction. It is necessary to account for elution volume (V_e) because only a portion of the extract is used in qPCR.

$$A)\ R_{f(2017)} = \frac{(X_d \times V_e)}{(X_l \times V_s)} \times 100$$

$$B)\ R_{s(2017)} = \left(\frac{M_i}{M_e}\right) \times \frac{(X_d \times V_e)}{(X_l \times V_s)} \times 100$$

$$C)\ R_{s(2018)} = \frac{(X_d \times V_e)}{(X_l \times V_s)} \times 100$$

Sample Matrix Trial

Initial optimization tests were conducted to quantify the DNA recovery from sediment and water samples. A total of twelve samples, including six sediment pellet and six supernatant

water were processed in triplicate. Six water samples containing suspended sediment were collected from field site DE-PA-11A. Five hundred milliliters of each field sample was distributed between 10, 50 ml falcon tubes while being stirred continuously using magnetic stir bar. Five microliters of freshly prepared *C. elegans* lysate (representing approximately $1.5x10^5$-$2.5x10^5$ *gfp* gene copies) were added to each of the 10 falcon tubes. The 50 ml tubes were shaken by hand for 10 s and centrifuged at 2,000 g for 10 min. Without disturbing the pellet, 100 ml of supernatant was filtered through 0.4 µm pore-size polycarbonate filters. Every field sample was filtered in triplicate. Filters were placed directly in GeneRite (North Brunswick, NJ) bead tubes and stored at -80 ˚C until DNA extraction. The mass of the soil pellets was recorded before freezing in -80°C before extraction. The soil pellets were extracted using the FastDNA™ SPIN Kit for Soil (MPBio, Santa Ana, CA) protocol using lysing matrix E homogenized for 40 sec at 6 m s^{-1}. All extractions were run in triplicate and were each eluted in 100ul of elusion buffer. All filter and pellet extracts were then processed using CG4 qPCR assay (Table 3) and the sample recoveries were calculated using Eq. S1 and S2 for filter and pellet extracts respectively. The percent recovery detected in sediment samples was greater than that of filtered samples (Fig S1). Field data from 2017 bolstered this trend and thus, only sediment samples was collected in 2018.

Lysing Matrix and Homogenization Time Trial

Another experiment was conducted to optimize the lysing matrix (bead type) and homogenization (bead beating) time was performed using sediment retrieved from site DE-PA-11A (Fig S2). Half a gram of sediment with five microliters of *C. elegans* (representing approximately $1.5x10^5$-$2.5x10^5$ *gfp* gene copies) lysate was added to each of the bead tubes

containing eight lysing matrices; A, C, D, E, F, G, H, and I (MPBio, Santa Ana, CA). One triplicate of lysing matrix tubes were homogenized at 6m s^{-1} for 40 s while another triplicate of the tubes were homogenized at $6ms^{-1}$ for 20 s. Extractions were performed using the FastDNA™ SPIN Kit for Soil (MPBio, Santa Ana, CA). Percent recovery was calculated using Eq. S2. Based on the results of this experiment (Fig S2), Lysing matrix C when homogenized for 20 s was selected to process all sediment samples in this study.

Artificial green fluorescent protein (*gfp*) gene sequence in C. elegans SH52 used design CG4 qPCR primers and probes

>
ATGAGTAAAGGAGAAGAACTTTTCACTGGAGTTGTCCCAATTCTTGTTGAATTAGATGGTGATGTTAATGGGC
ACAAATTTTCTGTCAGTGGAGAGGGTGAAGGTGATGCAACATACGGAAAACTTACCCTTAAATTTATTTGCACT
ACTGGAAAACTACCTGTTCCATGGGTAAGTTTAAACATATATATACTAACTAACCCTGATTATTTAAATTTTCA
GCCAACACTTGTCACTACTTTCTGTTATGGTGTTCAATGCTTCTCGAGATACCCAGATCATATGAAACGGCATG
ACTTTTTCAAGAGTGCCATGCCCGAAGGTTATGTACAGGAAAGAACTATATTTTTCAAAGATGACGGGAACTAC
AAGACACGTAAGTTTAAACAGTTCGGTACTAACTAACCATACATATTTAAATTTTCAGGTGCTGAAGTCAAGTT
TGAAGGTGATACCCTTGTTAATAGAATCGAGTTAAAAGGTATTGATTTTAAAGAAGATGGAAACATTCTTGGA
CACAAATTGGAATACAACTATAACTCACACAATGTATACATCATGGCAGACAAACAAAAGAATGGAATCAAAG
TTGTAAGTTTAAACATGATTTTACTAACTAACTAATCTGATTTAAATTTTCAGAACTTCAAAATTAGACACAAC
ATTGAAGATGGAAGCGTTCAACTAGCAGACCATTATCAACAAAATACTCCAATTGGCGATGGCCCTGTCCTTTT
ACCAGACAACCATTACCTGTCCACACAATCTGCCCTTTCGAAAGATCCCAACGAAAAGAGAGACCACATGGTCC
TTCTTGAGTTTGTAACAGCTGCTGGGATTACACATGGCATGGATGAACTATACAAATAG
