# Supplementary figures and images for: Development and validation of rapid environmental DNA (eDNA) detection methods for bog turtle (Glyptemys muhlenbergii)
Source: PLoS One. 2019 Nov 14;14(11):e0222883. doi: 10.1371/journal.pone.0222883 (PMC6855662; doi:10.1371/journal.pone.0222883)

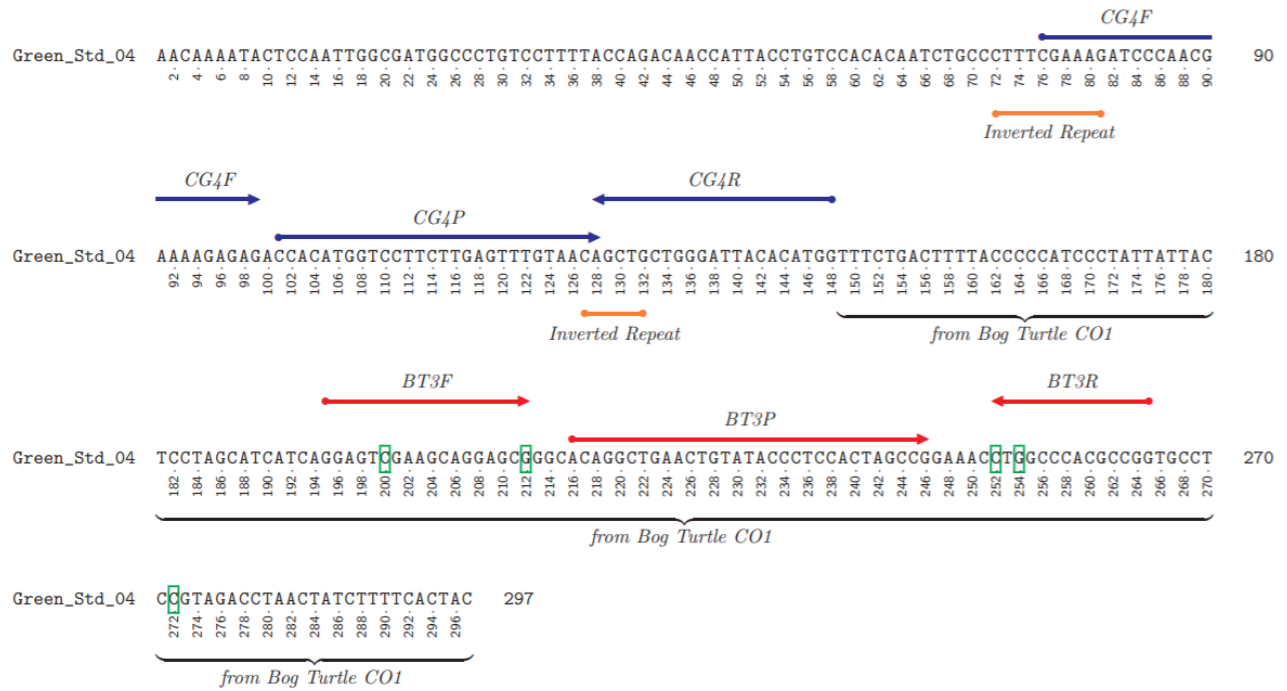

**S1 Fig. Oligonucleotide binding sites on gBlock used for standard curve generation.**

Supplement: S1 Fig — (PDF) [file pone.0222883.s003.pdf]

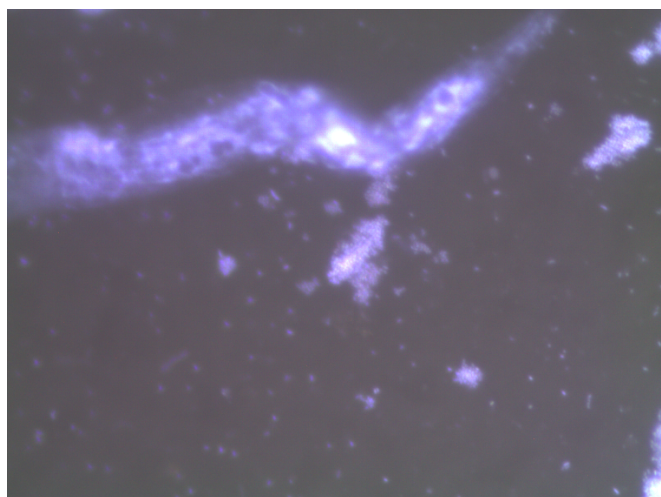

**S2 Fig. Micrograph of *C. elegans* lysate.**

Supplement: S2 Fig — Fluorescent micrograph of C. elegans lysate using DAPI stain (DNA-binding) showing intact cells and clumps of cells present in the lysate. Lysate used to spike in DNA extractions was filtered onto a 0.2 μm pore-size 25mm polycarbonate filter, stained, and viewed under a fluorescent microscope. The field of view is approximately 500 μm wide. A partially intact C. elegans individual appears near the top of the image. Cellular debris of various sizes can also be seen in the image indicating the bead-beating procedure does not completely lyse all tissues. (PDF) [file pone.0222883.s004.pdf]

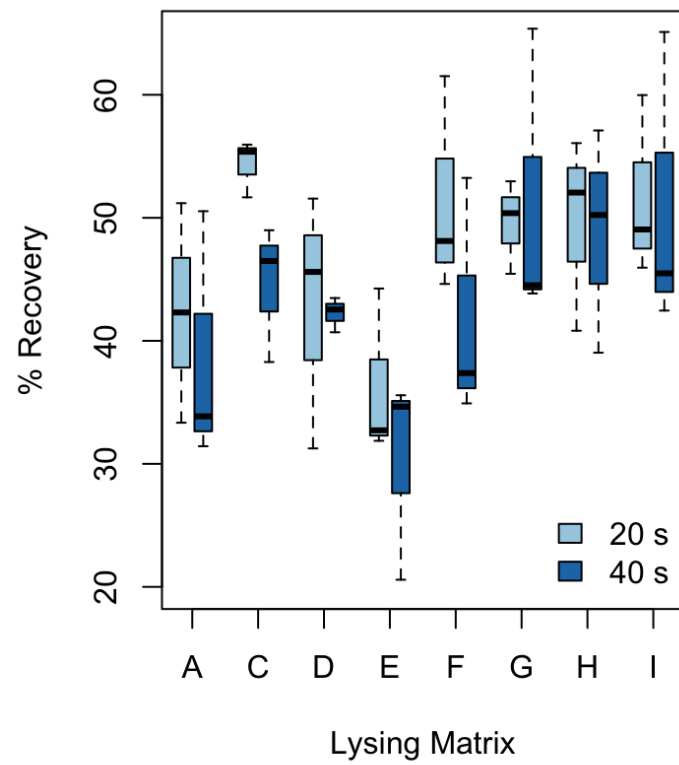

**S3 Fig. Results from lysing matrix and bead beating time optimization trial.**

Supplement: S3 Fig — Lysing matrix C was selected for its consistently high recovery with 20 s bead-beating. (PDF) [file pone.0222883.s005.pdf]

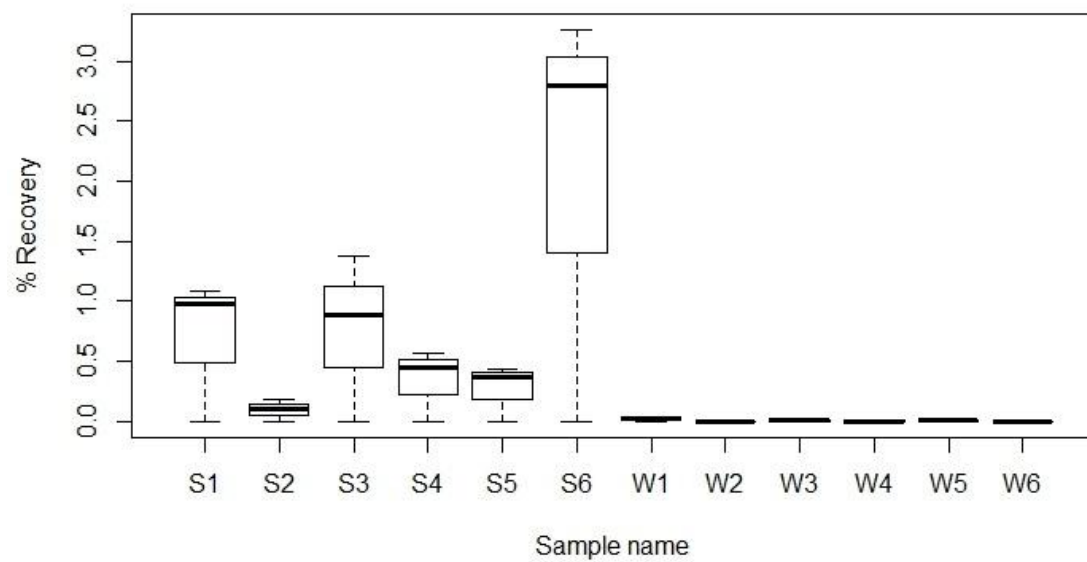

**S4 Fig. Results from sample matrix trial.**

Supplement: S4 Fig — Detectable DNA recovery from the sediment and supernatant water from field samples during extraction efficiency trials. “S1-S6” and “W1-W6” denote sediment and aqueous phases, respectively. (PDF) [file pone.0222883.s006.pdf]

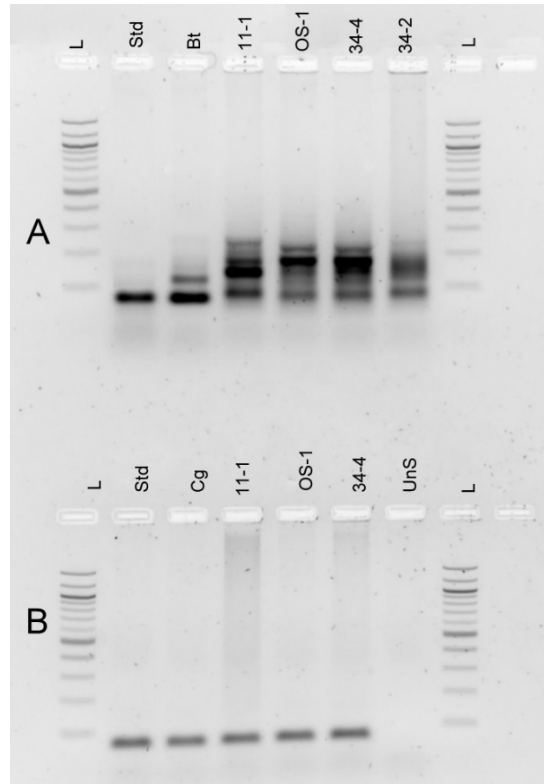

**S6 Fig. Gel image of non-specific amplification in BT3 assay.**

Supplement: S6 Fig — Gel electrophoresis of qPCR products from BT3 (A) and CG4 (B) on 2% agarose gel using 1X TBE buffer. L. 1kb Ladder; Std. gBlock standard; Bt. Bog turtle blood DNA; 11–1, OS-1, 34–4, 34–2. Environmental sediment sample DNA with C. elegans spike; Cg. C. elegans tissue DNA; UnS. Sediment with no C. elegans spike. (PDF) [file pone.0222883.s008.pdf]
