# Supplementary material for: Development and validation of rapid environmental DNA (eDNA) detection methods for bog turtle (Glyptemys muhlenbergii)
Source: PLoS One. 2019 Nov 14;14(11):e0222883. doi: 10.1371/journal.pone.0222883 (PMC6855662; doi:10.1371/journal.pone.0222883)
Supplement: S5 Fig — (PDF) [file pone.0222883.s007.pdf]

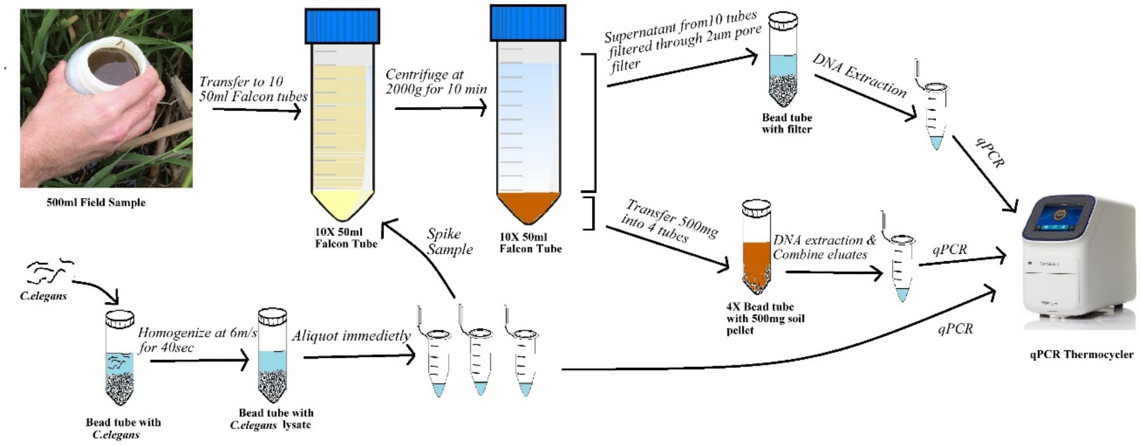

S5 Fig. Illustration of eDNA and *C. elegans* internal control processing workflow for 2017 environmental samples.
